# Changes in the lesion surface suggesting transformation of oral potentially malignant disorders to malignancy – a report of eight cases

**DOI:** 10.1186/s12903-023-02960-w

**Published:** 2023-05-09

**Authors:** Moon-Jong Kim, Hong-Seop Kho

**Affiliations:** 1 Department of Oral Medicine, Gwanak Seoul National University Dental Hospital, Seoul, South Korea; 2 Department of Oral Medicine and Oral Diagnosis, School of Dentistry and Dental Research Institute, Seoul National University, 101 Daehak-ro, Jongno-gu, Seoul, 03080 South Korea; 3 Institute on Ageing, Seoul National University, Seoul, South Korea

**Keywords:** Oral potentially malignant disorders, Malignancy, Transformation

## Abstract

**Background:**

In the case of oral potentially malignant disorders (OPMD), the possibility of malignant transformation of the lesion necessitates a decision on the need for an additional biopsy at each visit. Among many clinical characteristics, change on the lesion surface is one of the important factors that determine the need for additional biopsy at each visit. The purpose of the study was to provide information on the characteristics of lesions related to malignant transformation during the follow-up period of OPMD.

**Methods:**

Eight patients (four men and four women) with OPMD that transformed into malignancy during long-term follow-up were included and their mean age was 65.8 ± 12.4 years. Clinical information and histopathological diagnosis were investigated at the initial visit and during the long-term follow-up period. The focus was on information on changes on the lesion surface at the time the lesion was confirmed to be malignant. The period from initial diagnosis to dysplasia and from dysplasia to malignancy was also investigated.

**Results:**

The OPMD diagnoses were oral lichen planus or oral lichenoid lesions (n = 2), oral leukoplakia (n = 5), and hyperplastic candidiasis (n = 1). During the follow-up period of the lesions, when dysplasia was obtained by additional biopsy, changes in the lesions consisted of an increase in the size of the white or red area. The lesion surface of the OPMD showed verrucous, papillary, exophytic, corrugated, and ulcerative changes at the time of malignancy diagnosis. The period for the initial lesion to become dysplasia, from dysplasia to malignancy, and from the initial lesion to malignancy was very variable.

**Conclusions:**

Attention should be paid to verrucous, papillary, exophytic, corrugated, and ulcerative changes on the lesion surface of OPMD. Considering that the period for OPMD to become malignant is highly variable, a longer follow-up of the lesion is necessary.

**Supplementary Information:**

The online version contains supplementary material available at 10.1186/s12903-023-02960-w.

## Background

The complexity in treating oral mucosal diseases is due to the difficulty of diagnosis and prognosis prediction. In particular, in the case of oral potentially malignant disorders (OPMD), continuous decision making is required regarding the need for additional biopsy at every visit due to the potential of malignant transformation in the lesion. The most representative oral mucosal diseases with these characteristics include oral leukoplakia (OLK), oral lichen planus (OLP), oral lichenoid lesions (OLL), and hyperplastic candidiasis [1, 2].

OLK is an oral lesion defined as ‘a predominantly white plaque of questionable risk having excluded (other) known diseases or disorders that carry no increased risk for cancer’ [3]. OLK is a representative OPMD that should be monitored continuously for the possibility of malignant transformation [4–6]. OLP is a common immune-mediated oral mucosal disease, and it causes pain in the oral mucosa, makes it difficult to eat, and has a significant effect on the patient’s quality of life [7]. OLP is chronic, difficult to cure, and becomes malignant in about 1% of cases [8–13], so it is necessary for the clinician to determine the need for an additional biopsy at each patient visit. OLL is difficult to clearly distinguish from OLP using clinical examination only, but it is distinguished from OLP through comprehensive judgement of the clinical signs and histopathological findings [14, 15]. OLL includes oral lichenoid contact lesions, oral lichenoid drug reactions, and oral lichenoid lesion of graft-versus-host disease [16]. The transformation rate of OLL to malignancy is known to be higher than that of OLP [8, 10], so periodic observation of the lesion is mandatory. Hyperplastic type of candidiasis is the rarest form of oral candidiasis. Hyperplastic candidiasis has been called candida leukoplakia or leukocandidiasis, and the possibility of transformation into malignancy has been reported [17, 18].

Although there are reagents, devices or instruments, and molecular biological procedures that can be used to examine the potential of malignant transformation of OPMD [5, 19, 20], the reality is that most clinics still rely on clinical examination and biopsy. Therefore, there have been reports of clinical characteristics that enable the estimation of the risk for malignant transformation of OPMD. These characteristics include clinical characteristics such as age, gender, smoking status, site, size, colour, and shape of the lesion as well as histopathological features such as dysplasia [4–6, 21]. Among these, change on the lesion surface is one of the most important characteristics and one of the important factors that determine the need for additional biopsy at each visit.

The purpose of this study was to provide clinical information on cases that were not malignant at the time of initial examination but that transformed to malignancy during the follow-up period. The focus was on obtaining information on changes on the lesion surface at the time the lesion was confirmed to be malignant. The results of this study can provide essential information for the early detection of oral malignancy.

## Methods

### Subjects

The subjects were recruited from among patients with oral mucosal diseases who visited one doctor (HSK) at the Department of Oral Medicine, Seoul National University Dental Hospital from January 2006 to December 2015. Inclusion criteria were (1) the results of the clinical and histopathological examination at the time of initial examination were OPMD and (2) the oral lesion was transformed to malignancy during the long-term follow-up period. Eight patients (four men and four women) were included in the study, and the mean age (± SD) of the patients was 65.8 ± 12.4 years. The Institutional Review Board of Seoul National University Dental Hospital approved this study (#ERI22035) and authorised an exemption of informed consent from the subjects. All procedures were carried out in accordance with relevant guidelines and regulations.

### Clinical characteristics of subjects and oral lesions at the initial visit

All information was based on the electronic medical record (EMR), photographs of the lesion taken at the initial visit and periodically, and biopsy reports. There was no omission of clinical photographs of the lesion at the time of the initial visit and additional biopsy. We investigated information on clinical symptoms and duration, the main lesion related to the chief complaint, additional lesions in other oral mucosal areas, the period of recognition of the lesion, medical history, smoking status, and alcohol use in the past year at the time of the initial visit.

We investigated the colour, size, margin, surface, and clinical and histopathological diagnosis of the main lesion at the initial visit. When there were additional lesions in other oral mucosal areas, the clinical and histopathological diagnoses of the lesions were also investigated. The colour of the lesion was classified as white, red, or white and red; the lesion size as ≤ 1 cm^2^, > 1 cm^2^ ≤ 2 cm^2^, and > 2 cm^2^; and the margin of the lesion as distinct, intermediate, and indistinct. The surface of the lesion was classified as verrucous (or exophytic) or not. The colour, size, margin, surface, and clinical diagnosis of the lesion were determined by two authors (MJK and HSK) based on information obtained from the EMR and clinical photographs. When the opinions of the two authors differed, the two authors discussed and came to a consensus.

### Clinical history and histopathological changes of the main lesion

We investigated the reason for performing an additional biopsy of the main lesion, the histopathological diagnosis, and the interval at which the additional biopsy was performed. The change in the lesion at the time of the malignancy diagnosis was focused. The duration of the period from diagnosis at the initial visit to oral epithelial dysplasia (OED) and from OED to malignancy was also investigated.

## Results

### Oral symptoms and lesions and patient medical condition at the initial visit

Six of eight subjects complained of pain on spicy food, and the duration of symptoms was very variable (range, 1–66 months). Main lesions related to the chief complaint were present on the lateral or ventral surfaces of the tongue in five patients, two of whom had similar lesions found in other oral mucosal areas (both buccal mucosa in one patient, ventral surface of the contralateral side of the tongue in another). In the remaining three patients, the main lesion was located in the buccal mucosa and buccal alveolar mucosa, and the labial mucosa was included in one of these patients. The length of time that the patients were aware of the lesions was also very variable (1–181 months). Among the patients, two were smokers, two were ex-smokers, and four were non-smokers. Three of the patients were alcohol users in the past year, and the rest were not alcohol users (Table 1).

**Table 1:** Oral symptoms, lesions, and medical conditions of the patients at the initial visit

Patient No.	Sex	Age	Symptom		Lesion			Medical history and conditions	Smoking status	Alcohol use
			Type	Period (Mn)^a^	Main area	Other area	Period (Mn)^b^			
1	M	72	Pain on spicy food	2	Lateral and ventral area of Lt tongue	None	2	Tb (cured), hepatoma (cured), HT	Ex-smoker	No
2	M	61	Pain on spicy food	1	Lateral area of Lt tongue	Both buccal mucosa	181	HT (Mx), GERD (Mx)	Ex-smoker	Yes
3	M	71	No symptom		Lt lower buccal gingiva and Lt buccal mucosa	None	2.5	Endocarditis (Mx), HT (Mx), hyperlipidemia (Mx)	Non-smoker	Yes
4	F	68	Pain on spicy food	17	Lateral and ventral area of Lt tongue	None	17	Breast Ca (cured), thyroid Ca (cured), HT (Mx), hyperlipidemia (Mx), arthritis (Mx)	Non-smoker	No
5	F	39	No symptom		Lateral and ventral area of Lt tongue	Ventral area of Rt tongue	120	Allergic rhinitis	Non-smoker	No
6	F	80	Pain on spicy food	66	Rt upper labial and buccal alveolar mucosa	Lt upper labial alveolar mucosa	66	HT (Mx)	Smoker	No
7	M	62	Pain on spicy food	54	Lt buccal mucosa	Rt buccal mucosa	54	Tb (cured), HT (Mx), hearing problem	Smoker	Yes
8	F	73	Foreign body sensation	1	Ventral area of Lt tongue	None	1	None	Non-smoker	No

Ca, cancer; GERD, gastroesophageal reflux disease; HT, hypertension; Lt, left; Mn, months; Mx, under medications; Rt, right; Tb, tuberculosis ^a^Period from symptom recognition to initial clinic visit

^b^Period from recognition of the main lesion to initial clinic visit

### Clinical characteristics and clinical and histopathological diagnosis of oral lesions at the initial visit

Table 2 shows information on the main and other oral lesions at the initial visit. Most lesions were white and red mixed lesions (n = 6), and the size was greater than 2 cm^2^ (n = 5). The lesion margin was distinct in one case, and the lesion surface was verrucous or exophytic in three cases. One of two lesions clinically diagnosed as OLP or OLL and one of five lesions considered as OLK were OED histopathologically. The lesion considered epithelial hyperplasia due to denture irritation by clinical examination was found to be hyperplastic candidiasis on biopsy. Four patients had lesions in other oral mucosal areas along with the main lesion, all of whom had the same clinical diagnosis as the main lesion. In the three cases considered to be OLK, the main lesion was nonhomogeneous type (NH-OLK), and the other lesion was homogeneous type (H-OLK).

**Table 2:** Characteristics and diagnosis of the main and other oral lesions at the initial visit

Patient No.	Main lesion						Other lesions	
	Colour	Size	Margin^a^	Verrucous or exophytic	Diagnosis		Diagnosis	
					Clinical	Histopathological	Clinical	Histopathological
1	White and red	> 2 cm^2^	Indistinct	No	OLP or OLL	OLP	None	None
2	White and red	> 2 cm^2^	Indistinct	No	OLP or OLL	OED	OLP or OLL	Hyperkeratosis and acanthosis
3	White	> 1 cm^2^, ≤ 2 cm^2^	Intermediate	No	H-OLK	Hyperkeratosis and acanthosis	None	None
4	White	> 1 cm^2^, ≤ 2 cm^2^	Intermediate	No	H-OLK	Hyperkeratosis	None	None
5	White and red	> 2 cm^2^	Indistinct	No	NH-OLK	Hyperkeratosis and acanthosis	H-OLK	None
6	White and red	> 2 cm^2^	Intermediate	Verrucous	NH-OLK	OED	H-OLK	None
7	White and red	> 2 cm^2^	Intermediate	Exophytic	NH-OLK	Hyperkeratosis	H-OLK	None
8	White and red	> 1 cm^2^, ≤ 2 cm^2^	Distinct	Exophytic	Epithelial hyperplasia due to denture irritation	Chronic hyperplastic candidiasis	None	None

OED, oral epithelial dysplasia; H-OLK, homogeneous oral leukoplakia, NH-OLK, nonhomogeneous oral leukoplakia; OLL, oral lichenoid lesion; OLP, oral lichen planus

^a^Lesion margin: indistinct, not sharply demarcated margin; distinct, sharply demarcated margin; intermediate, between distinct and indistinct

### History for clinical and histopathological changes of main oral lesion

Table 3 shows the reasons for additional biopsy and their histopathological diagnoses for main lesions, and Table 4 shows the duration of the period from the diagnosis at the initial visit to malignancy. Figures 1, 2 and 3 and Figures S1 to S5 show the clinical photographs of the main lesions at the time of biopsy.

**Table 3:** Biopsy history of the main oral lesion

Patient No.	First biopsy	Second biopsy			Third biopsy			Fourth biopsy		
		Reason	Period (Mn)^a^	Dx	Reason	Period (Mn)^b^	Dx	Reason	Period (Mn)^c^	Dx
1	OLP (Fig. 1a)	Increase in white area	15	OED (Fig. 1b)	Surgical excision of lesion	10	OED	Verrucous change of lesion	50	SCC (Fig. 1c)
2	OED (Fig. S1a)	Verrucous change of lesion	7.5	SCC (Fig. S1b)						
3	Hyperkeratosis and acanthosis (Fig. 2a)	Increase in white area	20	Inflammation	Increase in lesion area	29	Inflammation and candidiasis (Fig. 2b)	Papillary change of lesion	5	SCC (Fig. 2c)
4	Hyperkeratosis (Fig. S2a)	Increase in red area	32	OED (Fig. S2b)	Papillary change of lesion	8	CIS (Fig. S2c)			
5	Hyperkeratosis and acanthosis (Fig. S3a)	Patient’s request	6.5	Hyperkeratosis and acanthosis	Ulceration of lesion surface	57	SCC (Fig. S3b)			
6	OED (Fig. S4a)	Increase in verrucous change of lesion	6	SCC (Fig. S4b)						
7	Hyperkeratosis (Fig. S5a)	Increase in exophytic change of lesion	7.5	CIS (Fig. S5b)						
8	Chronic hyperplastic candidiasis (Fig. 3a)	Recurrence of exophytic lesion	6	Hyperkeratosis and inflammation	Recurrence of exophytic lesion	5	Candidiasis (Fig. 3b)	Corrugated change of lesion surface	15	SCC (Fig. 3c)

CIS, carcinoma-in-situ; Dx, diagnosis; OED, oral epithelial dysplasia; Mn, months; OLP, oral lichen planus; SCC, squamous cell carcinoma ^a^Period from the first to second biopsies. ^b^Period from the second to third biopsies. ^c^Period from the third to fourth biopsies

**Table 4:** Period for histopathological changes in the main oral lesion

Patient No.	Period based on biopsy (Mn)			Period based on patient’s history (Mn)
	From hyperkeratosis to OED	From OED to SCC or CIS	From hyperkeratosis to SCC or CIS	From lesion recognition to SCC or CIS
1	15^a^	60 (SCC)	75^b^ (SCC)	77 (SCC)
2		7.5 (SCC)		187.5 (SCC)
3			54 (SCC)	56.5 (SCC)
4	32	8 (CIS)	40 (CIS)	57 (CIS)
5			63.5 (SCC)	183.5 (SCC)
6	2^c^	6 (SCC)	8 (SCC)	72 (SCC)
7			7.5 (CIS)	61.5 (CIS)
8			26^d^ (SCC)	27 (SCC)

CIS, carcinoma-in-situ; Mn, months; OED, oral epithelial dysplasia; SCC, squamous cell carcinoma

^a^Period from oral lichen planus to OED.

^b^Period from oral lichen planus to SCC.

^c^Period based on referral document containing the previous biopsy report

^d^Period from chronic hyperplastic candidiasis to SCC.

**Fig. 1:**
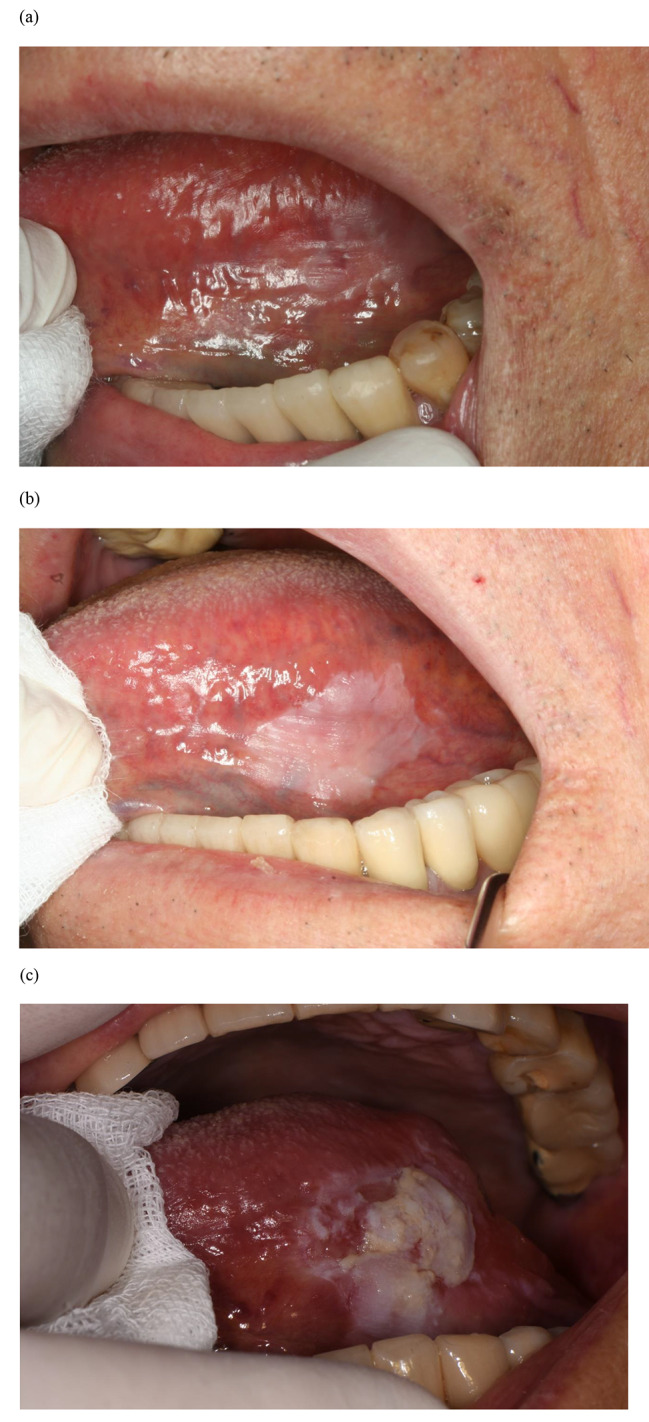
Case No. 1. (a) White and red lesion on the left lateral and ventral surfaces of the tongue at the initial visit. Histopathological diagnosis was oral lichen planus. (b) Lesion 15 months after the initial visit. The increase in the white area was a reason for another biopsy. Histopathological diagnosis was oral epithelial dysplasia. (c) Lesion 75 months after the initial visit. The verrucous change in the lesion was a reason for additional biopsy. Histopathological diagnosis was squamous cell carcinoma

**Fig. 2:**
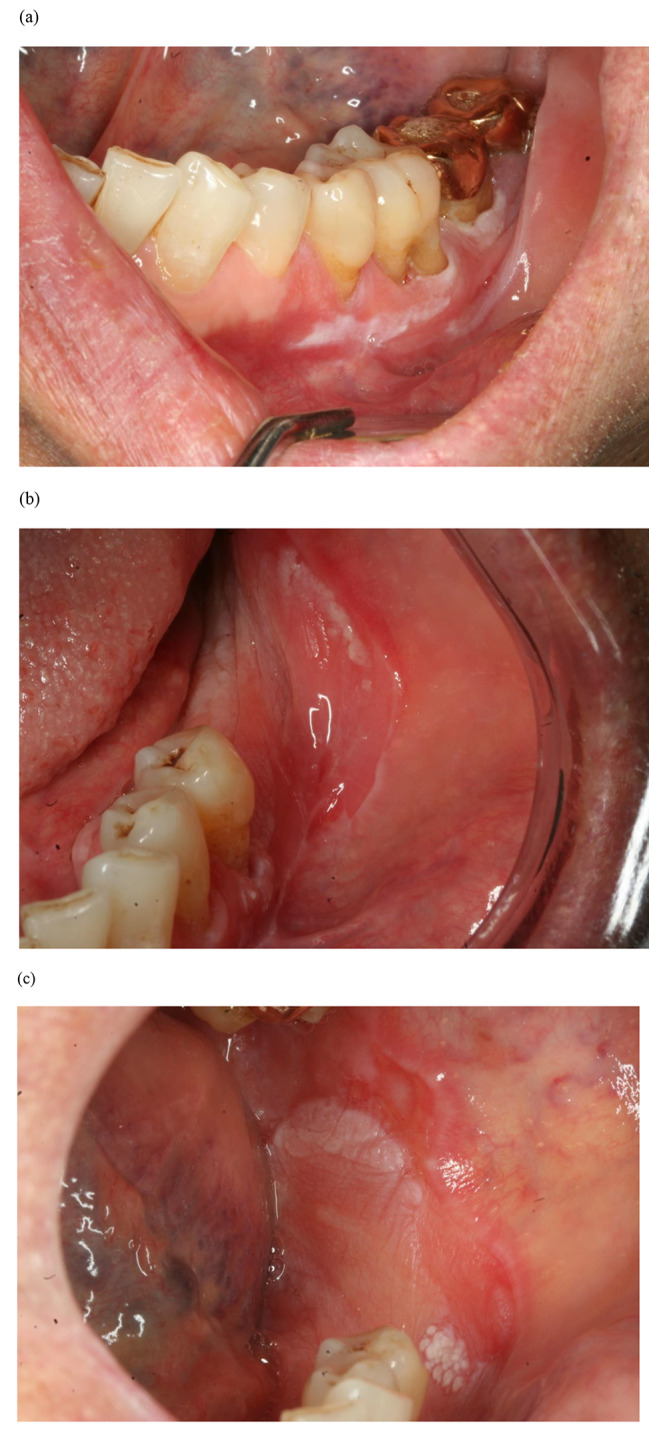
Case No. 3. (a) White lesion on the left lower buccal gingiva at the initial visit. Histopathological diagnosis was hyperkeratosis and acanthosis. (b) Lesion 49 months after the initial visit. The increase in the lesion into buccal mucosa was a reason for additional biopsy. Histopathological diagnosis was inflammation and candidiasis. (c) Lesion 54 months after the initial visit. The papillary change in the lesion was a reason for additional biopsy. Histopathological diagnosis was squamous cell carcinoma

**Fig. 3:**
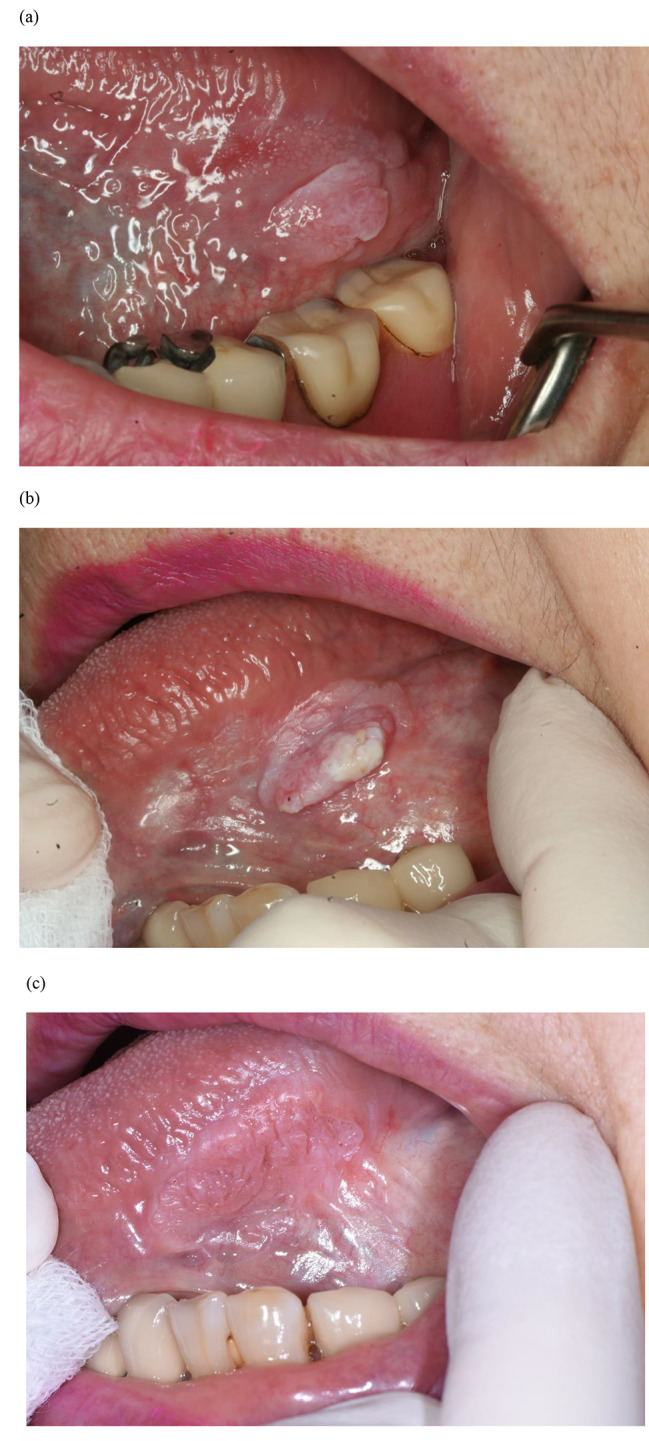
Case No. 8. (a) White and red exophytic lesion on the left ventral surface of the tongue at the initial visit. Histopathological diagnosis was hyperplastic candidiasis. (b) Lesion 11 months after the initial visit. The recurrence of exophytic lesion was a reason for additional biopsy. Histopathological diagnosis was candidiasis. (c) Lesion 26 months after the initial visit. The corrugated change in the lesion was a reason for additional biopsy. Histopathological diagnosis was squamous cell carcinoma

During the follow-up period of the lesions, when OED was obtained by additional biopsy, changes in the lesions consisted of an increase in the size of the white or red area (No. 1 and 4; Table 3; Fig. 1b and S2b). Considering these two cases as well as another case (No. 6) confirmed by the biopsy report from the referral hospital, the duration of the period from OLP or hyperkeratosis to OED was very variable (2, 15, and 32 months, respectively; Tables 3 and 4).

At the time of malignancy diagnosis of the lesions that were transformed from OED to malignancy (n = 4, No. 1, 2, 4, and 6), all lesions showed verrucous changes on the lesion surface (Table 3; Fig. 1c, S1b, S2c, and S4b). In one case (No. 4), the term *papillary* was more appropriate than *verrucous* (Table 3 and Figure S2c). The duration of the period from OED to malignancy was also very variable (6–60 months; Table 4).

At the time of malignancy diagnosis of the lesions showing transformation from hyperkeratosis to malignancy (n = 3, No. 3, 5, and 7), the changes on the lesion surface were papillary (Fig. 2c), ulceration (Figure S3b), and exophytic (Figure S5b). In these three cases, the duration of transformation from hyperkeratosis to malignancy was 7.5, 54, and 63.5 months, respectively. In the case of hyperplastic candidiasis showing transformation to malignancy (No. 8), the change in the lesion at the time of malignancy diagnosis was a corrugated change on the lesion surface (Fig. 3c). The duration of the period of transformation to malignancy was 26 months (Tables 3 and 4).

In all five cases of OLK (No. 3–7) showing transformation from hyperkeratosis to malignancy, with or without a diagnosis of OED, multifocal verrucous changes suggesting proliferative verrucous leukoplakia having a higher risk were not found during the follow-up period. The duration of transformation from hyperkeratosis to malignancy ranged from 7.5 to 63.5 months. There was no clear difference between NH (7.5, 8, and 63.5 months) and H (40 and 54 months) types (Tables 2 and 3, and 4).

Based on patient history, the duration of the period from the time the patient recognised the lesion to malignancy was 77 and 187.5 months in the clinical diagnosis of OLP or OLL, 56.5–183.5 months in OLK, and 27 months in hyperplastic candidiasis (Table 4).

## Discussion

The main purpose of this study was to provide the changes on the lesion surface in detail that occur during the course of the progression of OPMD lesions to malignancy.

In the present study, cases of OPMD were OLP or OLL, OLK, and hyperplastic candidiasis. OLP or OLL and OLK have been considered OPMD, and there are many previous reports on the transformation rate of these lesions into malignancy [4–6, 8–13]. Hyperplastic candidiasis was not included in the recent classification of OPMD and the reason for the exclusion was given [2]. However, the results of the present study suggest that further discussion is needed.

When treating patients with OPMD, the most important question is the malignancy potential of the lesion. Therefore, a variety of information that can estimate the risk of OPMD transformation into malignancy is important. In particular, information on clinical characteristics is very useful because it can be applied routinely during long-term management. The risk factors of OPMD transformation to malignancy are suggested to be age, female gender, non-smoking status, tongue and floor of the mouth, red colour, relatively large size, sharply demarcated margin, nonhomogeneous texture, and proliferative characteristics [4–6]. In most of the cases in the present study, the site, colour, and size of the lesion at the initial visit showed high-risk characteristics. All patients except one were over 60 years of age, and half of the patients, including the 39-year-old patient, were females. Half of the patients were non-smokers and three of the five patients with OLK were of the nonhomogeneous type. Therefore, risk factors such as age, female gender, non-smoking status, and nonhomogeneous texture were also somewhat congruent. However, results of the margin and surface characteristics of the lesion did not show high-risk characteristics in most cases at the time of the initial visit, which suggests that it is difficult to determine the possibility of malignant transformation based on the lesion characteristics at the initial visit. Although a biopsy procedure is required, the presence of dysplasia has been reported as the most powerful predictor of malignant transformation [6]. OED was found at the initial visit or during the follow-up in four of the eight cases in the present study.

A key question in the present study was about the changes on the lesion surface associated with malignant transformation. Our results showed that the lesion surface change related to OED, a powerful predictor of malignant change, was an increase in the size of white or red lesions. Because there may be an increase or decrease in lesion size during long-term follow-up, the change in the lesion size alone cannot be considered as a decisive factor. When the OED was transformed to malignancy, the changes in the lesion surface were verrucous in all four cases. In one of these cases, the term *papillary* rather than *verrucous* was more appropriate for describing the change on the lesion surface. Three cases of hyperkeratosis-to-malignant transformation showed papillary, ulcerative, and exophytic lesion surface changes in association with malignant transformation. In a case of hyperplastic candidiasis, the corrugated lesion surface change was associated with malignant transformation. Thus, the results of the present study suggest that attention should be paid to verrucous, papillary, corrugated, exophytic, and ulcerative changes during follow-up of OPMD lesions.

Although the number of cases was small in the present study, the results of the study indicated that the periods from the initial lesion to OED, from OED to malignancy, and from the initial lesion to malignancy were too variable, consistent with the results of previous studies. The estimated mean (± SD) of evolution time of OLK until malignant transformation reported by combining the results of ten studies was 3.2 ± 0.9 years, with a range between 1.8 and 5.1 years [6]. That of OLP and OLL was 8 to 168 months [10]. Considering the results of the present study, which suggest that it may take more than 15 years from the time the patient recognises the lesion to the transformation to malignancy, a longer follow-up period is necessary for OPMD.

## Conclusions

The verrucous, papillary, corrugated, exophytic, and ulcerative changes in the lesion surface were showed at the time when OPMD transformed to malignancy. Therefore, attention should be paid to these changes in the lesion surface during the follow-up period of patients with OPMD. Because the highly variable duration of the period for OPMD to become malignancy, a longer period of follow-up of the lesion is required.

## Electronic supplementary material

Below is the link to the electronic supplementary material.

Supplementary Material 1

## Data Availability

All data generated or analysed during this study are included in this published article (and its supplementary information files).

## List of abbreviations

CIS: carcinoma-in-situ
EMR: electronic medical record
H-OLK: homogeneous oral leukoplakia
NH-OLK: nonhomogeneous oral leukoplakia
OED: oral epithelial dysplasia
OLK: oral leukoplakia
OLL: oral lichenoid lesions
OLP: oral lichen planus
OPMD: oral potentially malignant disorders
SCC: squamous cell carcinoma
