# Supplementary material for: Changes in the lesion surface suggesting transformation of oral potentially malignant disorders to malignancy – a report of eight cases
Source: BMC Oral Health. 2023 May 9;23:268. doi: 10.1186/s12903-023-02960-w (PMC10170730; doi:10.1186/s12903-023-02960-w)
Supplement: Supplementary file 1 — Supplementary Material 1 [file 12903_2023_2960_MOESM1_ESM.docx]

(a)


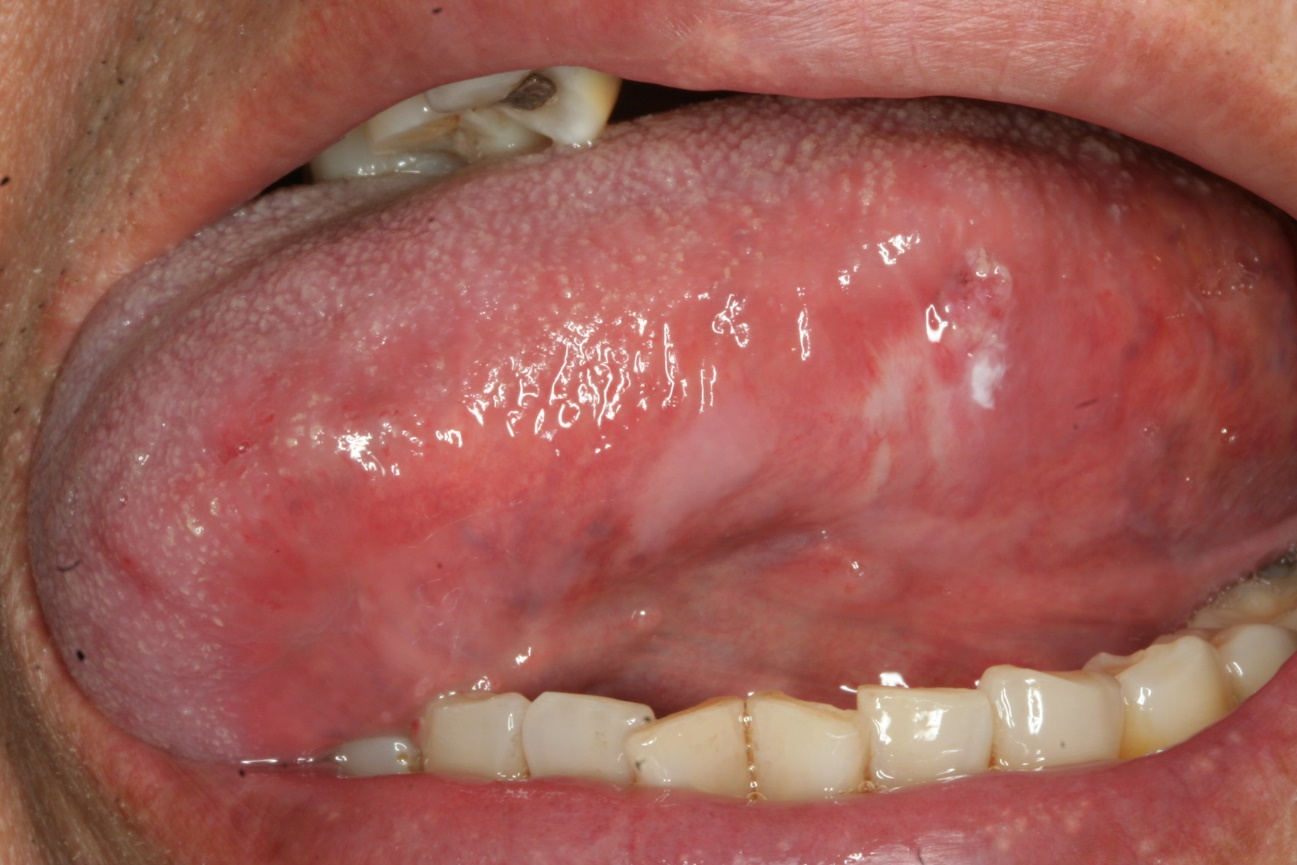


(b)


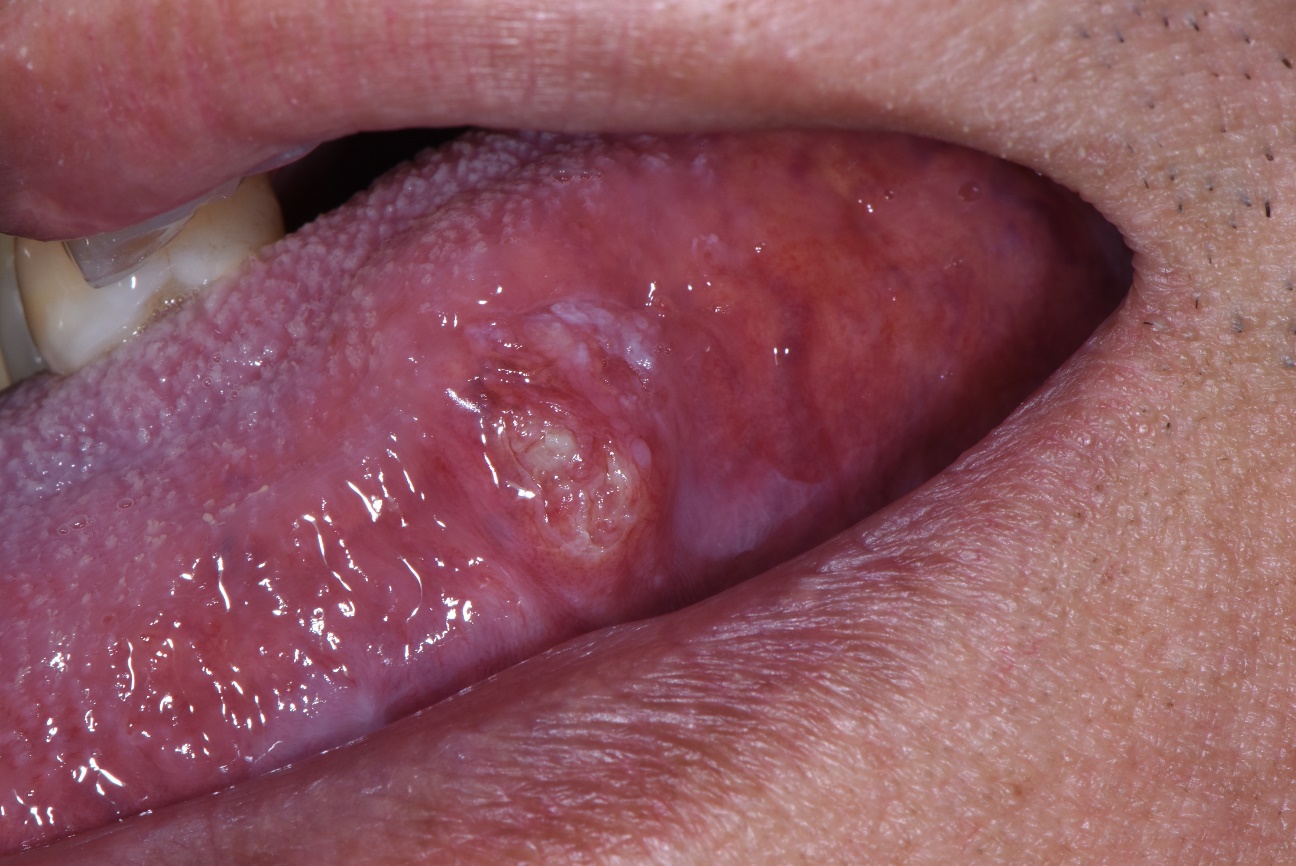


**Supplementary Figure 1.** Case No. 2. (a) White and red lesion on the left lateral surface of the tongue at the initial visit. Histopathological diagnosis was oral epithelial dysplasia. (b) Lesion 7.5 months after the initial visit. The verrucous change in the lesion was a reason for additional biopsy. Histopathological diagnosis was squamous cell carcinoma.

(a)


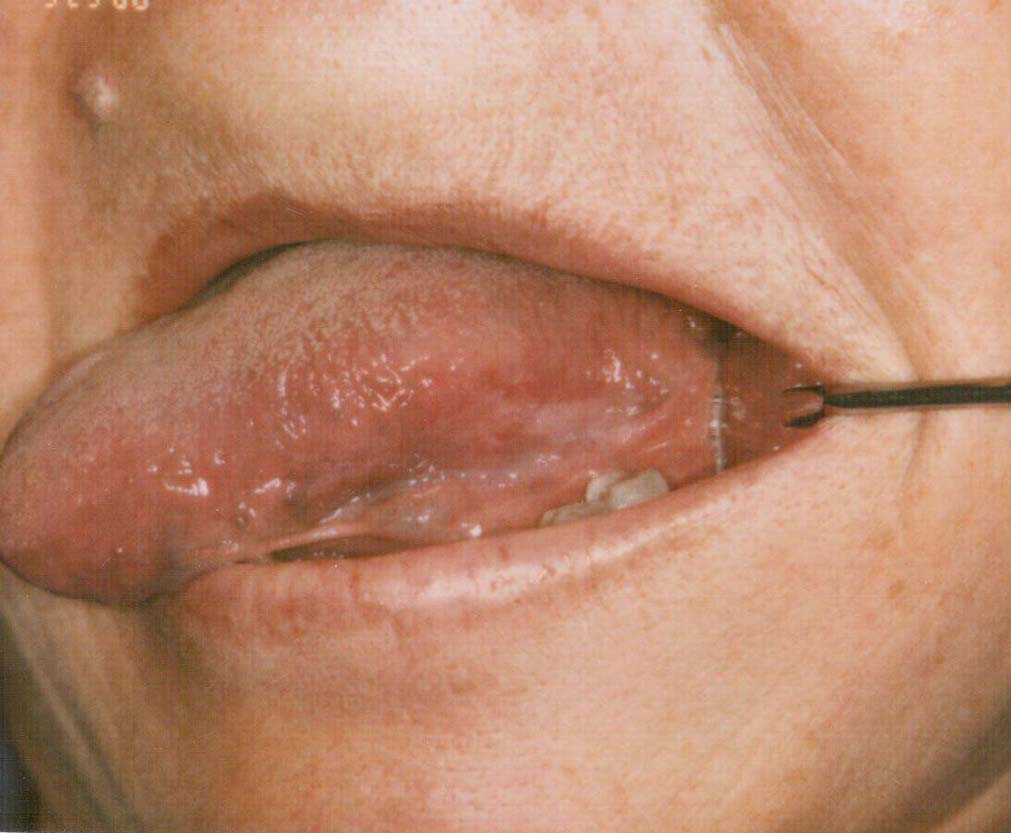


(b)


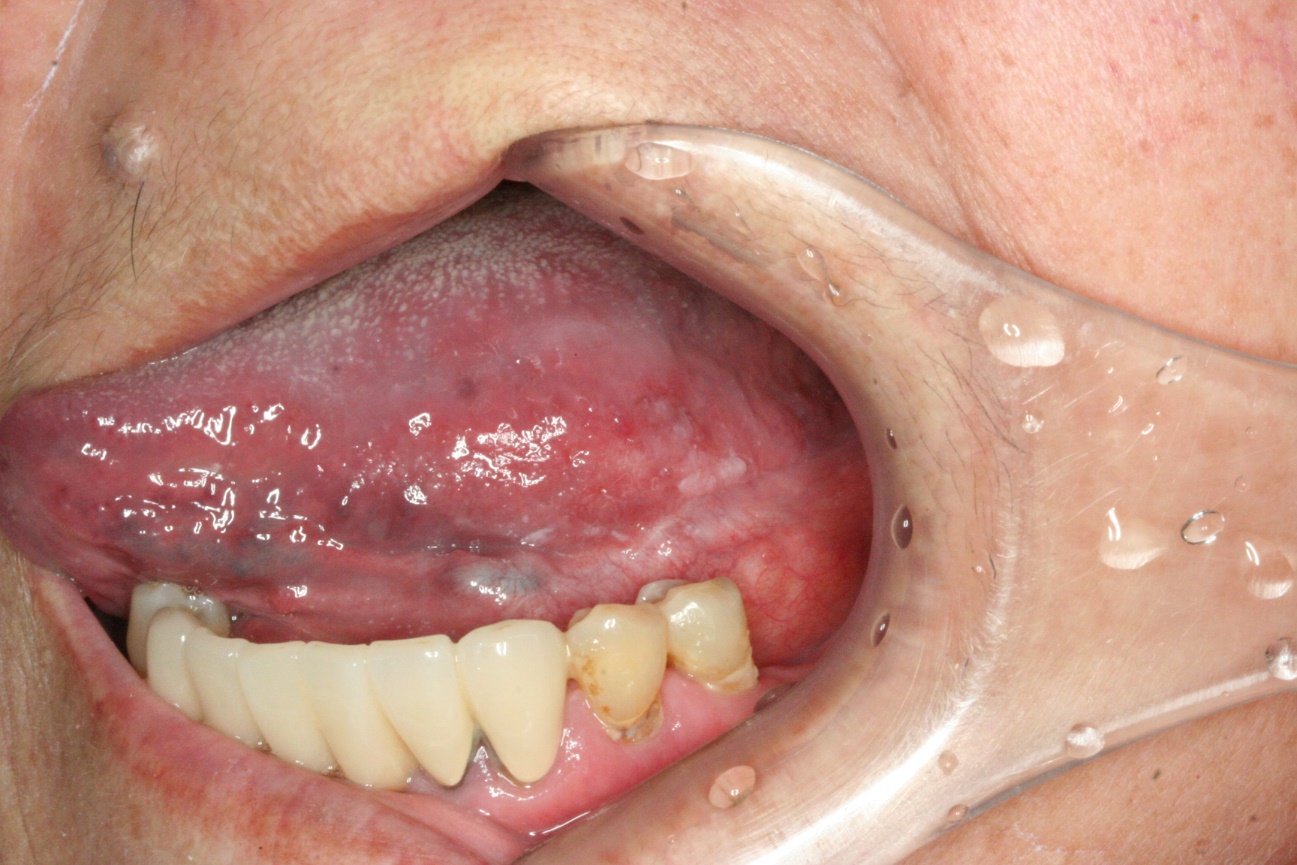


(c)


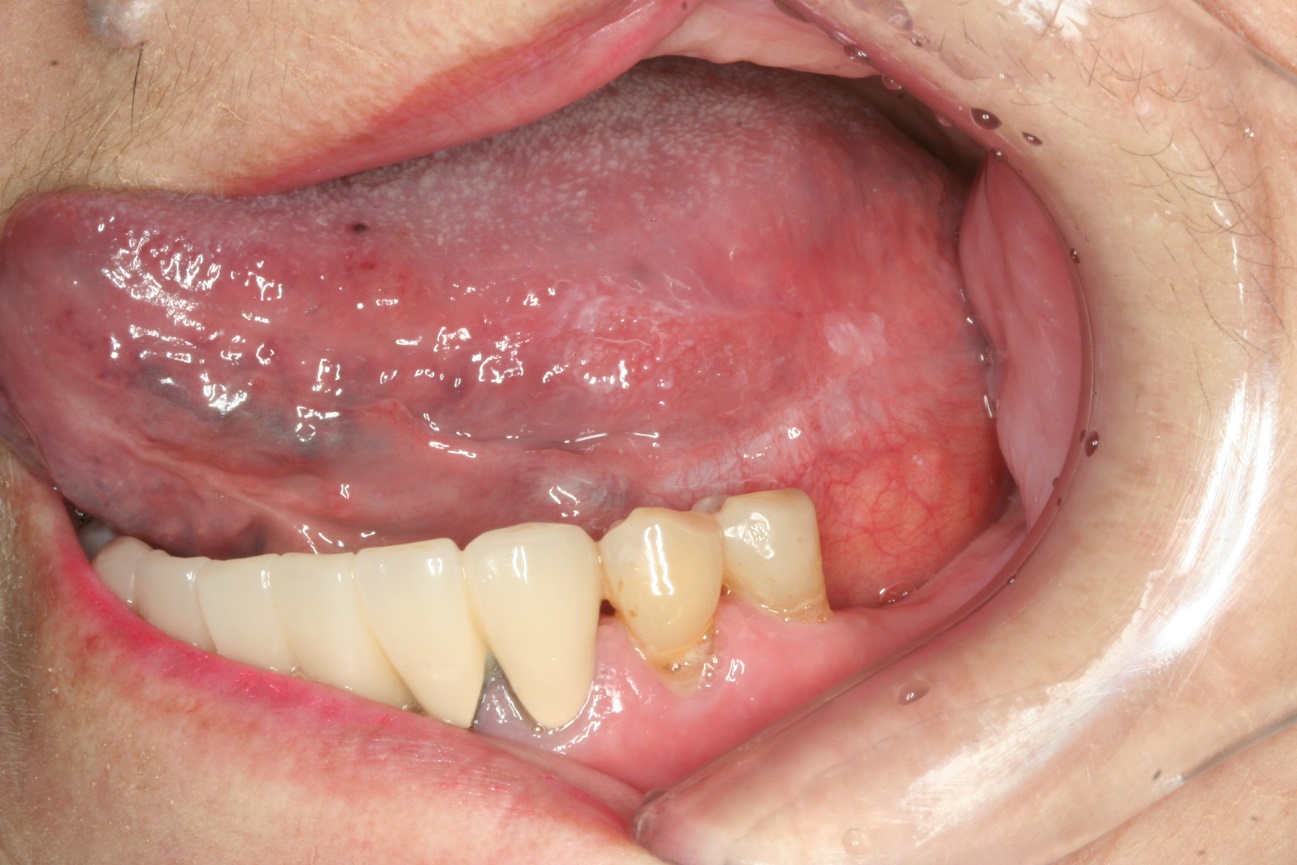


**Supplementary Figure 2.** Case No. 4. (a) White lesion on the left lateral and ventral surfaces of the tongue at the initial visit. Histopathological diagnosis was hyperkeratosis. (b) Lesion 32 months after the initial visit. The increase in the red area was a reason for another biopsy. Histopathological diagnosis was oral epithelial dysplasia. (c) Lesion 40 months after the initial visit. The papillary change in the lesion was a reason for additional biopsy. Histopathological diagnosis was carcinoma-in-situ.

(a)


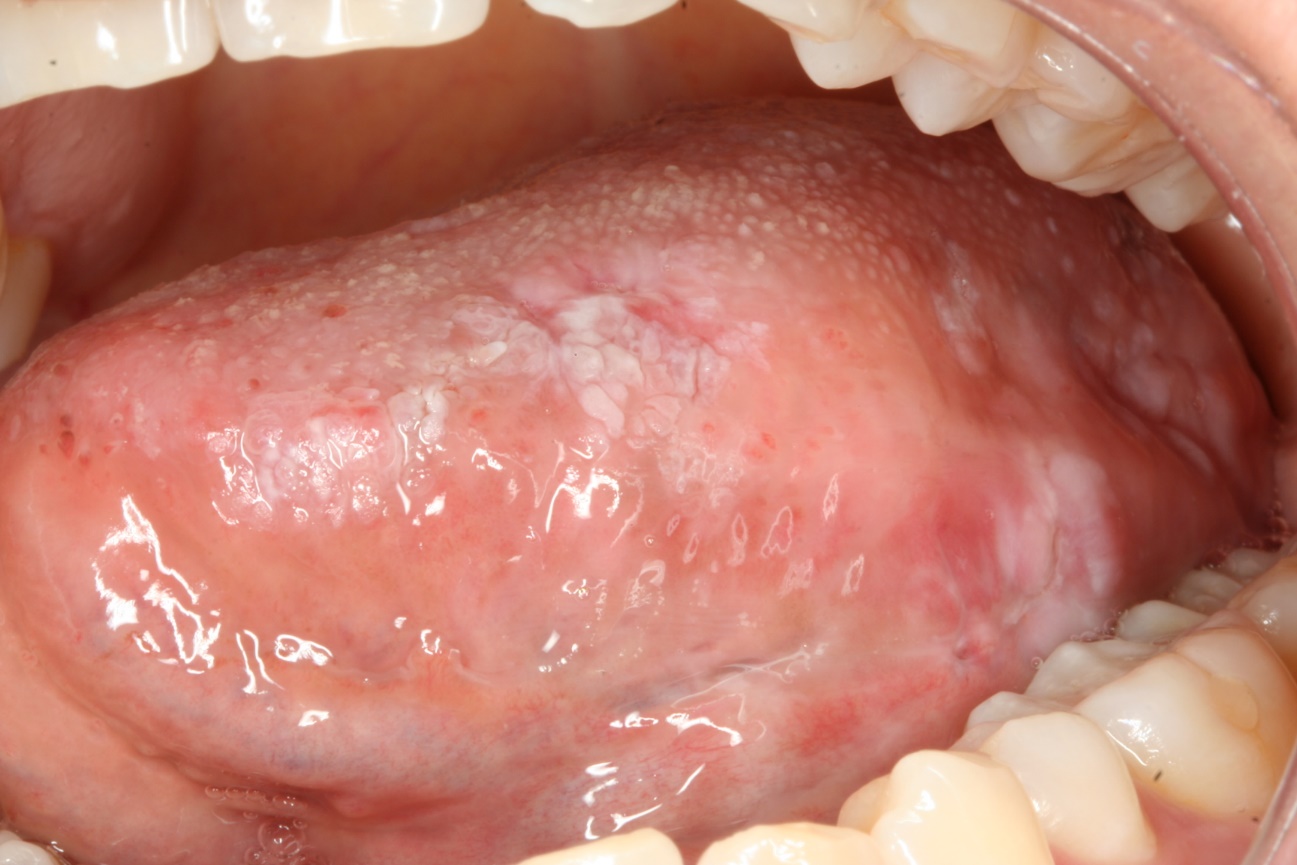


(b)


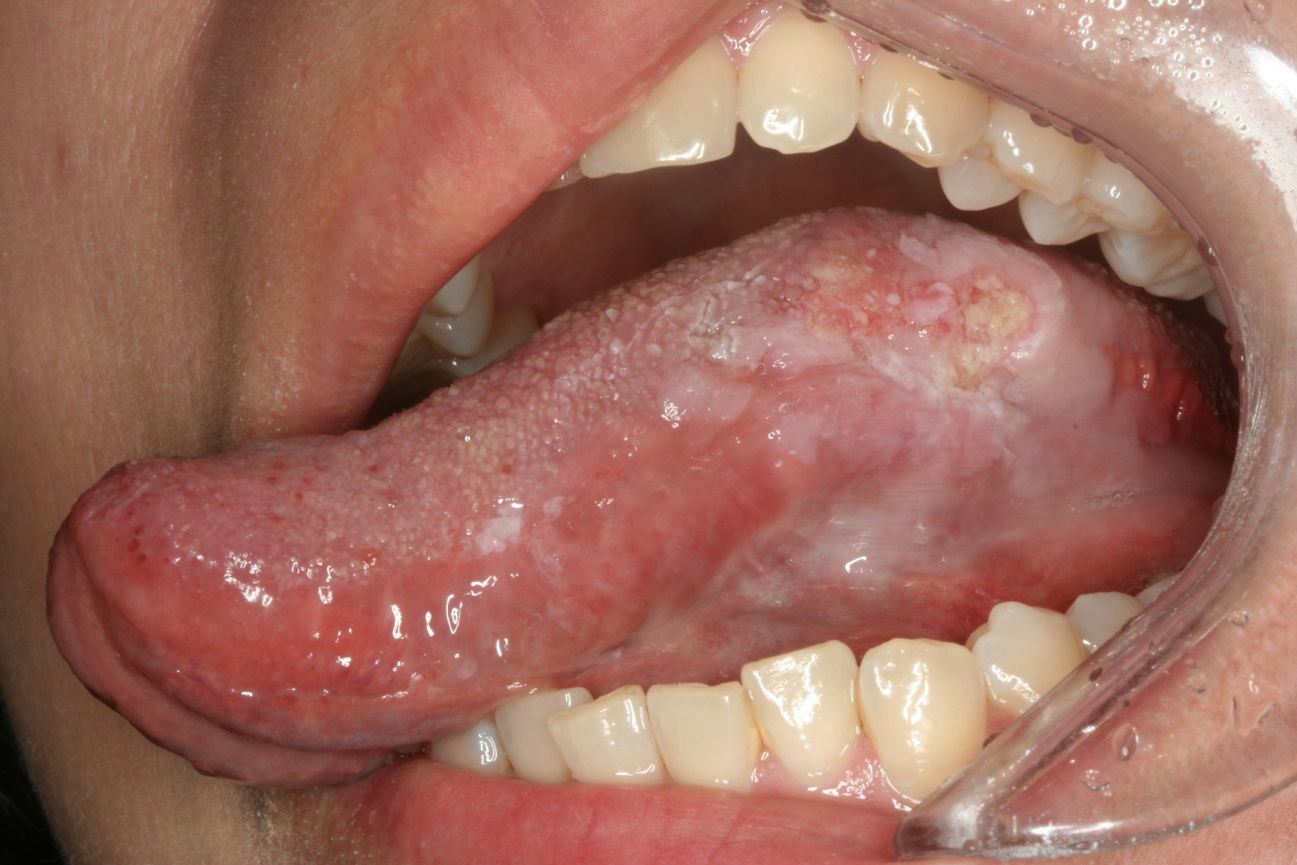


**Supplementary Figure 3.** Case No. 5. (a) White and red lesion on the left lateral and ventral surfaces of the tongue at the initial visit. Histopathological diagnosis was hyperkeratosis and acanthosis. (b) Lesion 63.5 months after the initial visit. The ulcerative change in the lesion was a reason for additional biopsy. Histopathological diagnosis was squamous cell carcinoma.

(a)


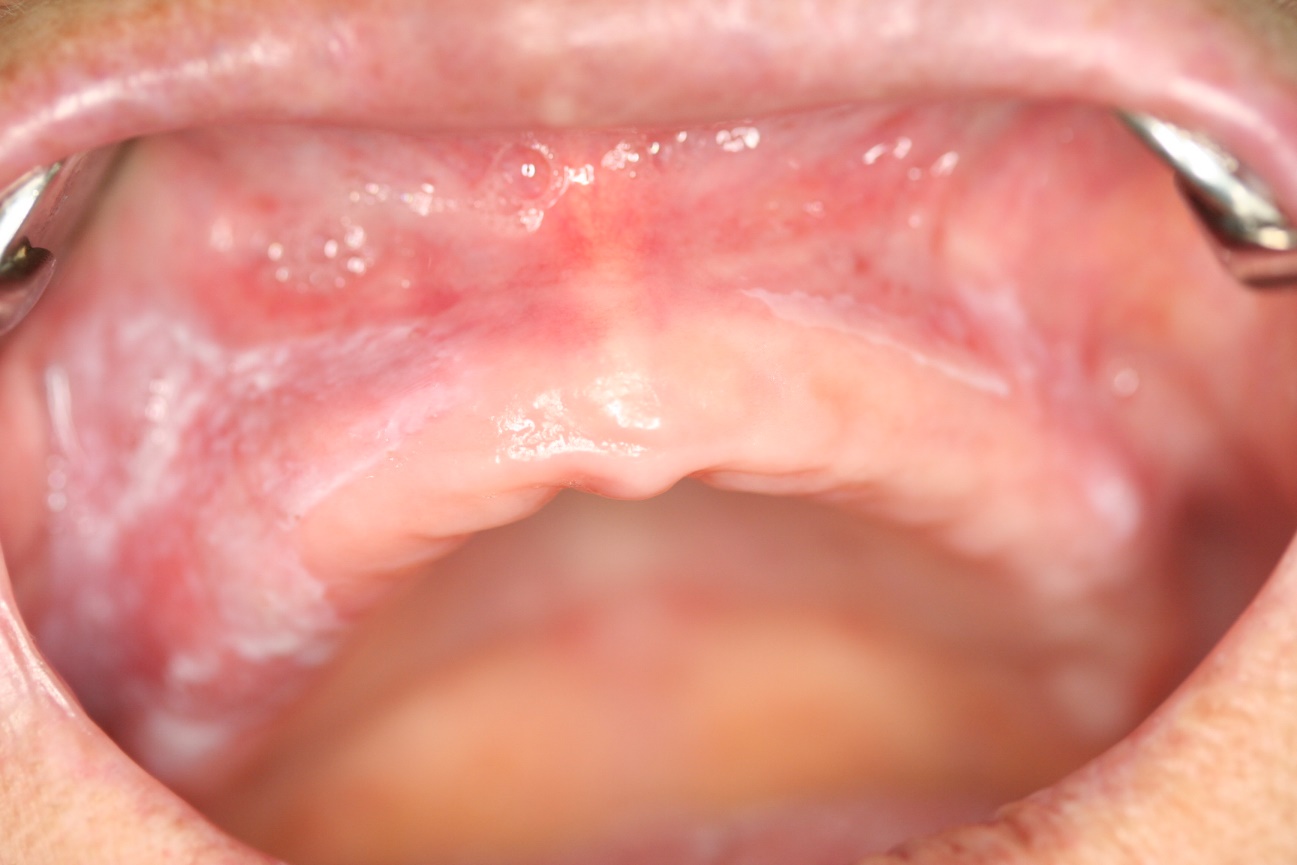


(b)


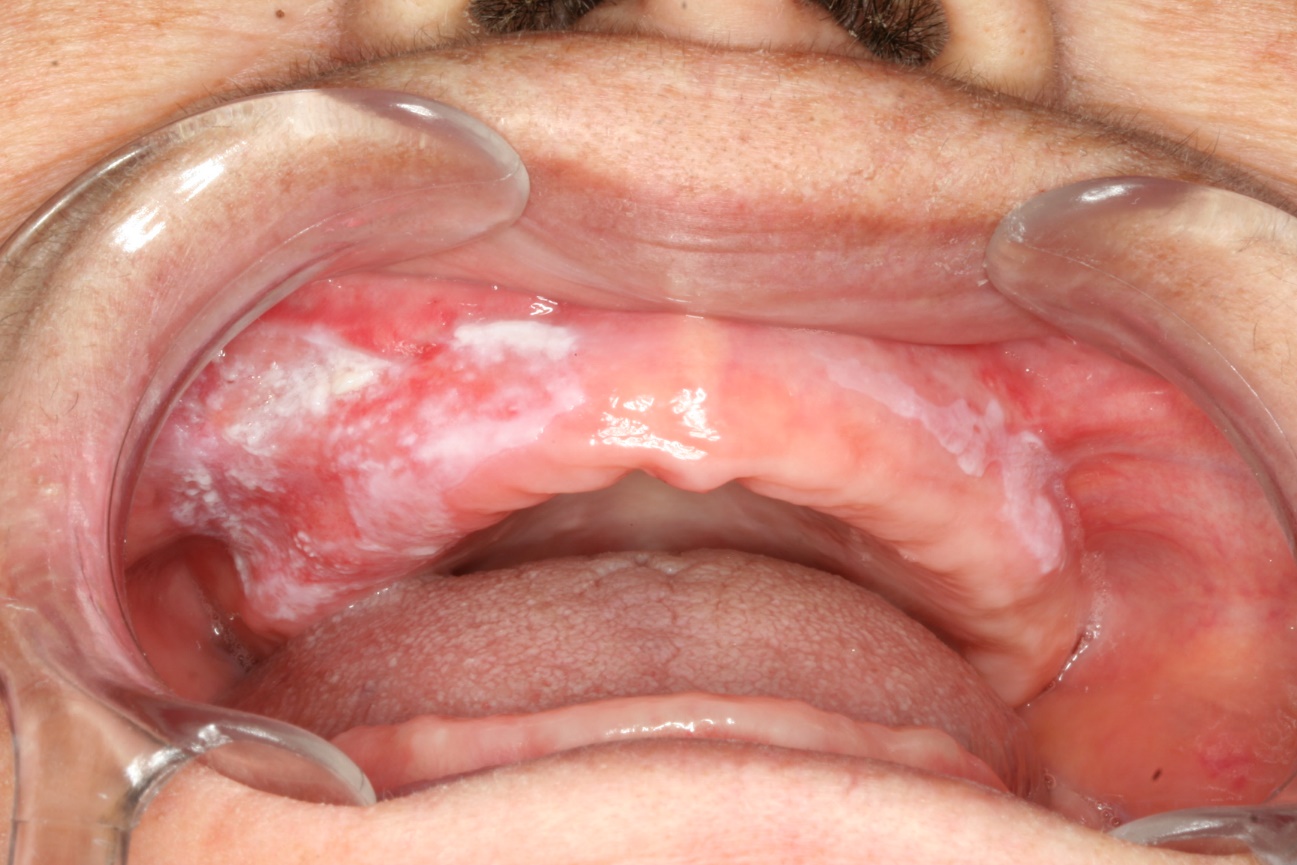


**Supplementary Figure 4.** Case No. 6. (a) White and red verrucous lesion on the upper labial and buccal alveolar mucosa at the initial visit. Histopathological diagnosis was oral epithelial dysplasia. (b) Lesion 6 months after the initial visit. The increased verrucous change of the right lesion was a reason for additional biopsy. Histopathological diagnosis was squamous cell carcinoma.

(a)


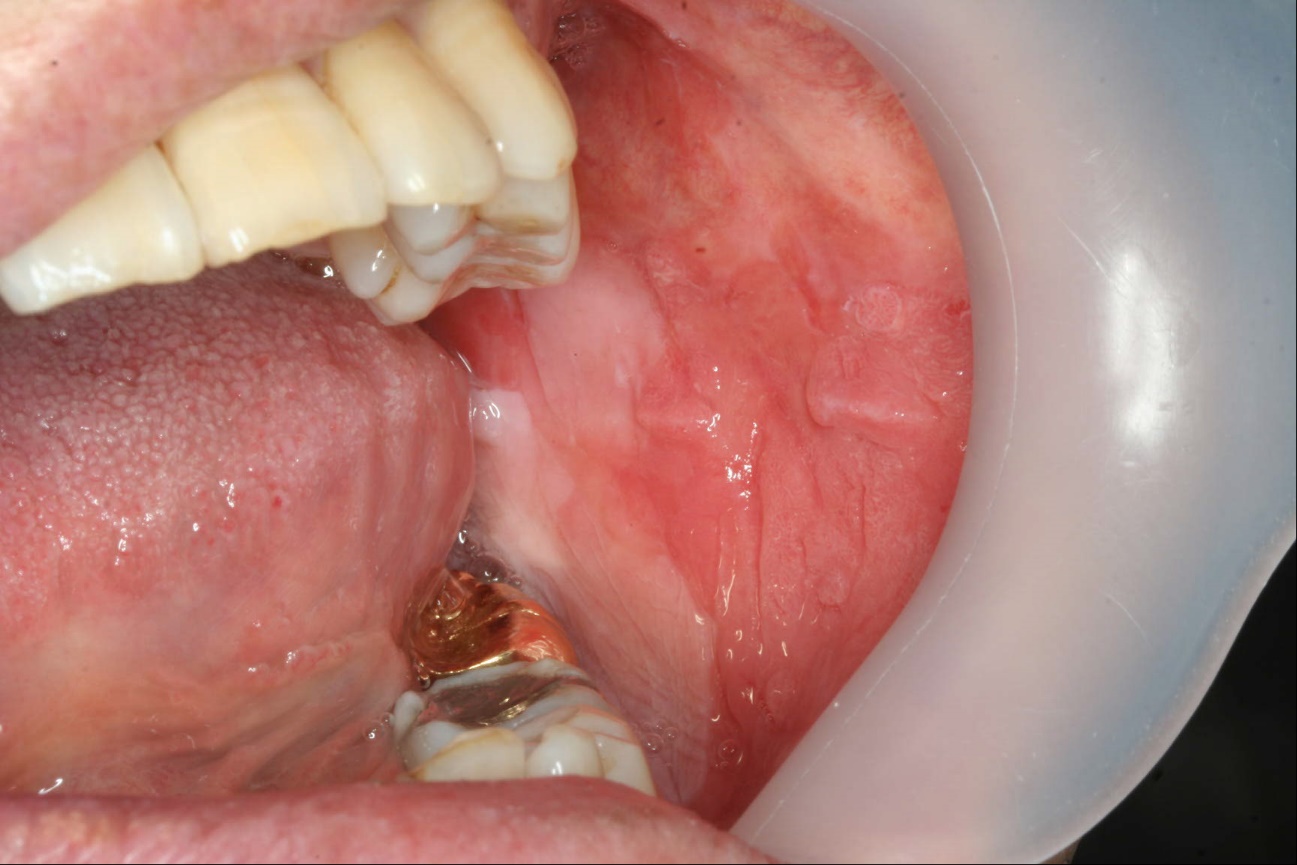


(b)


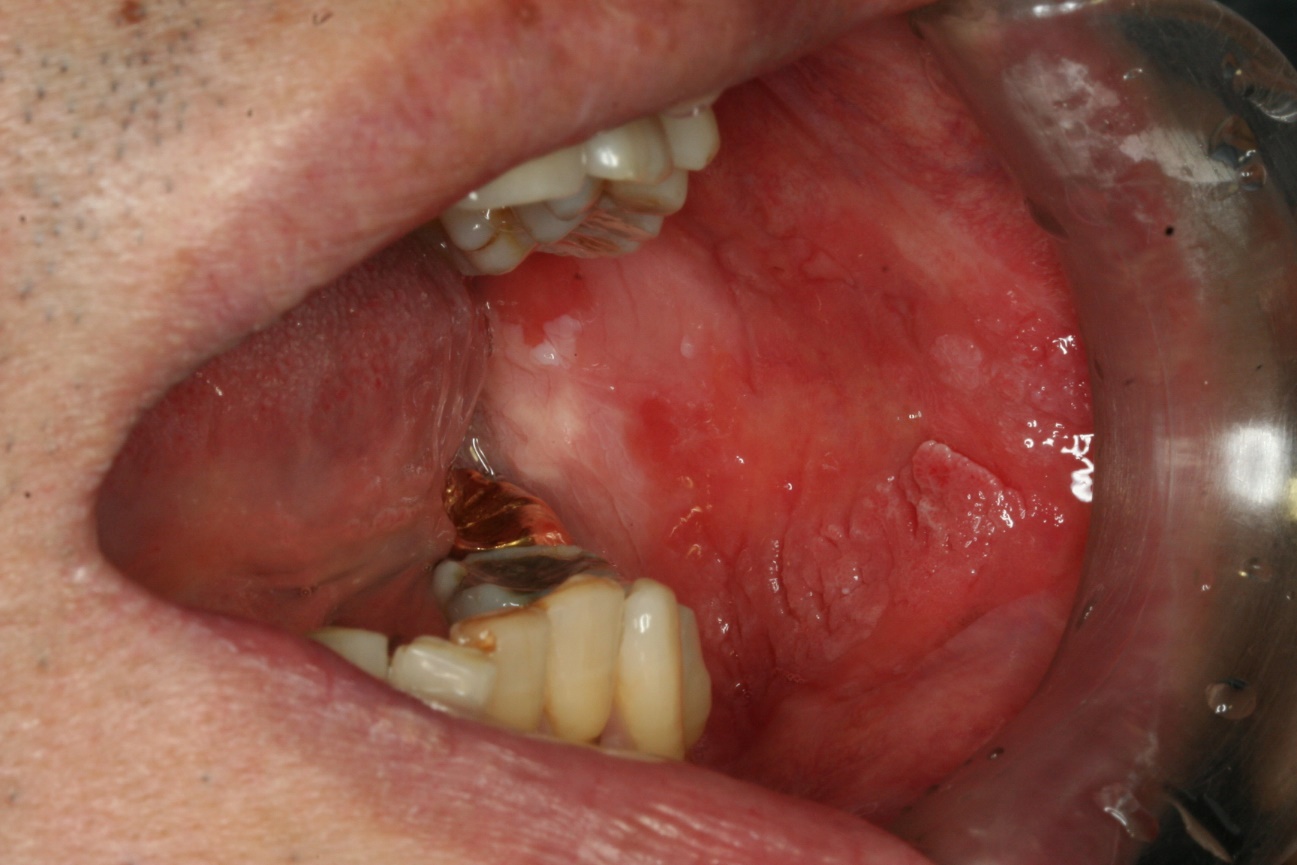


**Supplementary Figure 5.** Case No. 7. (a) White and red exophytic lesion on the left buccal mucosa at the initial visit. Histopathological diagnosis was hyperkeratosis. (b) Lesion 7.5 months after the initial visit. The increased exophytic change in the lesion was a reason for additional biopsy. Histopathological diagnosis was carcinoma-in-situ.
